# Canine and Equine Mesenchymal Stem Cells Grown in Serum Free Media Have Altered Immunophenotype

**DOI:** 10.1007/s12015-015-9638-0

**Published:** 2015-12-05

**Authors:** Kaitlin C. Clark, Amir Kol, Salpi Shahbenderian, Jennifer L. Granick, Naomi J. Walker, Dori L. Borjesson

**Affiliations:** Veterinary Clinical Sciences Department, University of Minnesota, Saint Paul, MN 55108 USA

**Keywords:** Equine, Canine, Mesenchymal stem cells, Immunomodulation, Lymphocyte, Bone marrow, Adipose tissue, Cell culture, Proliferation, Surface protein phenotype

## Abstract

Mesenchymal stem cell (MSC) therapy is being increasingly used to treat dogs and horses with naturally-occurring diseases. However these animals also serve as critical large animal models for ongoing translation of cell therapy products to the human market. MSC manufacture for clinical use mandates improvement in cell culture systems to meet demands for higher MSC numbers and removal of xeno-proteins (i.e. fetal bovine serum, FBS). While serum-free media (SFM) is commercially available, its affects on MSC phenotype and immunomodulatory functions are not fully known. The objective of this study was to determine if specific MSC culture conditions, MSC expansion in HYPERFlasks® or MSC expansion in a commercially available SFM, would alter MSC proliferation, phenotype or immunomodulatory properties in vitro. MSCs cultured in HYPERFlasks® were similar in phenotype, proliferative capacity and immunomodulatory functions to MSCs grown in standard flasks however MSC yield was markedly increased. HYPERFlasks® therefore provide a viable option to generate greater cell numbers in a streamlined manner. Canine and equine MSCs expanded in SFM displayed similar proliferation, surface phenotype and inhibitory effect on lymphocyte proliferation in vitro. However, MSCs cultured in the absence of FBS secreted significantly less PGE_2_, and were significantly less able to inhibit IFNγ secretion by activated T-cells. Immunomodulatory functions altered by expansion in SFM were species dependent. Unlike equine MSCs, in canine adipose-derived MSCs, the inhibition of lymphocyte proliferation was not principally modulated by PGE_2_. The removal of FBS from both canine and equine MSC culture systems resulted in altered immunomodulatory properties in vitro and warrants further investigation prior to moving towards FBS-free culture conditions.

## Introduction

Adult-derived multipotent mesenchymal stem cells (MSCs) hold great appeal for regenerative medicine therapeutic approaches in both human and veterinary medicine given their potent immunomodulatory and pro-regenerative properties [1, 2]. MSCs are defined in vitro by their morphology, their expression of a panel of cell surface markers and their potential to tri-lineage differentiate in vitro into adipose, bone and cartilage [3]. MSCs can be harvested from numerous tissue sources including bone marrow and adipose tissue followed by ex vivo expansion of isolated cells [2]. MSCs are being investigated for their potential clinical application in multiple disorders including orthopedic injuries and inflammatory/immune-mediated diseases [2, 4]. A deeper understanding of canine and equine MSC biology is critical to guide veterinary clinical trials as well as to assure appropriate translational research into naturally occurring diseases that could inform human clinical trials [5–7].

MSCs are traditionally expanded as an adherent monolayer in medium supplemented with fetal bovine serum (FBS). The Food and Drug Administration (FDA) requires that MSC products meet good manufacturing practice (GMP) and encourages the use of xeno-protein free culture conditions to decrease the potential for xeno-protein immune reactions and transmission of infectious diseases [8]. In addition, previous studies in human patients suggest that the development of anti-FBS antibodies in patients after MSC administration may be associated with attenuated clinical efficacy [9, 10]. FBS also has high lot-to-lot variability, which makes reproducibility extremely difficult [11–13]. Human MSCs maintain differentiation and proliferative capacities in xeno-protein free culture conditions, however there are also reports of induced senescence in vitro [11, 14–19]. The use of xeno-protein free culture conditions for canine and equine MSCs does not alter osteogenic and adipogenic differentiation potential, but did lead to inferior MSC proliferation [13]. The effect of serum free media (SFM) on canine and equine MSC immunomodulatory functions has not yet been determined.

In addition to the concerns regarding the use of FBS, there is an increasing need for the expansion of even greater MSC numbers for clinical applications [20]. MSC culture in small tissue-culture flasks is inefficient and requires a significant amount of time and reagents in order to achieve the required cell numbers for treatment. Large bioreactors permit the expansion of greater absolute MSC numbers, while greatly reducing reagent costs and technician time [21, 22]. HYPERFlasks® are multilayer flasks that use gas permeable chambers coated with CellBIND® to optimize cell binding. However, the CellBIND® coating is a novel proprietary substance, and its effects on canine and equine MSC proliferative capacity and immunomodulatory properties have not been investigated.

The objectives of this study were to investigate the effects of xeno-free, and CellBIND® (HYPERFlasks®) culture conditions on canine and equine MSC functions in vitro compared to standard culture conditions. We hypothesized that MSC expansion in SFM or HYPERFlasks® would not alter canine or equine MSC proliferation, surface phenotype or immunomodulatory properties in vitro. We found that SFM and CellBIND® did not alter MSC proliferation or surface phenotype and MSCs cultured in both conditions were effective in inhibiting T-cell proliferation in vitro. However MSCs cultured in SFM displayed significant alterations in mediator secretion patterns. Our study suggests that while the use of CellBIND® coated HYPERFlasks® are suitable for MSC culture upscale, the use of commercially available SFM needs closer investigation prior to clinical implementation.

## Materials and Methods

### Animal Cells

#### Equine

Low passage (P2-P5) bone marrow-derived MSCs (eMSCs) from 5 horses were obtained from the UC Davis (UCD) William R. Pritchard Veterinary Medical Teaching Hospital Regenerative Medicine Laboratory. These samples were originally submitted for MSC expansion for autologous patient treatment. Excess eMSCS not used for treatment were donated for research purposes with written owner consent.

#### Canine

Low passage (P2-P5) fat-derived MSCs from 5 dogs were obtained from falciform fat collected from UCD William R. Pritchard Veterinary Medical Teaching Hospital patients undergoing routine abdominal surgery. Fat was collected under an approved Institutional Animal Care and Use Committee and the Clinical Trials Review Board protocol at UCD. Fat was processed and canine adipose-derived MSCs (cMSCs) were isolated, expanded and cryopreserved exactly as previously described [23, 24].

### MSC Culture and Expansion

Cryopreserved MSCs were thawed in a 37 °C water bath and seeded into 1 of 3 different culture conditions. The first group consisted of standard culture conditions [25, 26]. These cells were plated at a density of 5000 cells/cm^2^ into T185 flasks (Thermo Fisher Scientific, Waltham, MA) in the standard medium [Dulbecco’s Modified Eagle’s Medium (DMEM, Gibco, Invitrogen, Carlsbad, CA) with 10 % FBS (HyClone, Logan, UT) and 1 % penicillin-streptomycin (Gibco). The second group consisted of MSCs plated in HYPERFlasks® (Corning Inc., Tewksbury, MA). These cells were initially thawed in standard conditions and were then passed into HYPERFlasks® at 5000 cells/cm^2^ in standard medium. The third group consisted of MSCs cultured in SFM. MSCs do not readily adhere to plastic in the absence of serum. Therefore, in the third group, MSCs were thawed into flasks (Thermo Fisher Scientific) that had been coated with bovine fibronectin (25–50 μg/mL, Biomedical Technologies Inc., Stoughton, MA) for 24 h after which cells were replated at a density of 5000 cells/cm^2^ in StemPro® MSC SFM (Gibco) supplemented with 1 % L-glutamine (Gibco) and 1 % Pen/Strep (Gibco). All MSCs were grown to 70 % confluence in a standard cell culture incubator conditions (37 °C, 5 % CO_2_). During expansion, MSCs were monitored daily for morphologic alterations. Cells cultured under standard conditions and in HYPERFlasks® were dissociated using 0.05 % trypsin/EDTA (Gibco) and neutralized with standard media containing FBS. MSCs grown in SFM were harvested using HyQTase (HyClone), and resuspended in Dulbecco’s Phosphate Buffered Saline (DPBS, Gibco).

### MSC Proliferation

Equine MSCs and cMSCs were harvested after 4 days of culture for proliferation studies. At day 4, MSCs were roughly 70 % confluent, and were counted using an automated cell counter (COULTER® A^c^T-Diff Cell Counter, Beckman Coulter Inc., Miami, FL). Cell doubling times were calculated as previously reported [26]. Absolute numbers were also recorded and normalized for differences in flask surface area in each culture condition.

### MSC Phenotype

#### Equine

After harvesting, MSCs expanded in each of the three conditions were washed and incubated with antibodies directed against CD90 (VMRD, Pullman, WA, clone DH24A) [27, 28], CD44 (AbD Serotec, Raleigh, NC, clone CVS18) [29], CD29 (Beckman Coulter Inc., clone 4B4LDC9LDH8) [29], F6B (a pan leukocyte antibody, a generous gift from Dr. Jeffrey Stott, UCD, School of Veterinary Medicine) [30], CD86 (BD Biosciences, Franklin Lakes, NJ, clone IT2.2) [28], MHC I (AbD Serotec; clone CVS22) [31], and MHC II (AbD Serotec; clone CVS20) [28].

#### Canine

After harvesting, MSCs expanded in each of the 3 conditions were washed and incubated with antibodies directed against CD90 [23], CD45 [23], MHCII (all from Leukocyte Antigen Biology Laboratory, UCD, School of Veterinary Medicine, clone CA1.4G8, CA12.10C12, CA2.1C12 respectively) [23], CD34 (BD Biosciences, clone RAM34) [23], CD54 (a generous gift from C. Smith, Houston, TX, clone CL18.1D8) [23] and CD44 (R&D Systems, Minneapolis, MN, clone 69-S5).

Equine MSC and cMSC phenotype were evaluated via flow cytometry (Cytomics, Beckman Coulter FC500, Hialeah, FL). Flow cytometry data was analyzed using FlowJo flow cytometry software (Tree Star Inc., Ashland, OR).

### Mixed Leukocyte Reaction (MLR)

#### Equine

Peripheral blood was collected into tubes containing acid–citrate dextrose (ACD; BD Biosciences) via jugular venipuncture. T lymphocytes were enriched from peripheral blood mononuclear cells (PBMCs) using nylon wool and plated exactly as previously described [27]. To inhibit MSC proliferation, MSCs were irradiated (10Gy, Varian 2100C linear accelerator, Varian Medical Systems Inc., Palo Alto, CA) and kept on ice before plating in each corresponding medium. Enriched T-cells were stimulated with phytohemagglutin at 5 μg/mL (PHA, Sigma-Aldrich, St. Louis, MO). Enriched T-cells from donor horses and allogeneic MSCs were plated exactly as previously described [27, 32].

#### Canine

Peripheral blood was collected into tubes containing sodium heparin (Vacutainer®, BD Biosciences) via jugular venipuncture*.* PBMCs were obtained using a discontinuous Ficoll gradient and were plated with allogeneic cMSCs exactly as previously described [24]. PBMCs were used for these assays in canines due to the large blood volume necessary to isolate lymphocytes using a nylon wool gradient. PMBCs were activated with 5 μg/mL concanavalin A (Con-A, Sigma-Aldrich).

After 3 days of co-culture, wells were treated with 1 mM Bromodeoxyuridine (BrdU, BD Biosciences). Twenty-four hours post BrdU treatment, leukocytes were collected and cells were stained for nuclear BrdU incorporation per manufacturer directions (FITC BrdU Flow Kit, BD Biosciences) and read by flow cytometry.

At the time of leukocyte collection, culture supernatant was collected, centrifuged and stored at −80 °C, as previously described [27] for the measurement of secreted mediators. The following ELISA kits were used: equine PGE_2_ (Prostaglandin E2 Parameter Assay Kit; R&D Systems) [33], canine PGE_2_ (Prostaglandin E2 Express EIA kit (Monoclonal); Cayman Chemical Company, Ann Arbor, MI) [24], equine interleukin10 (IL-10; Equine IL-10 Duoset; R&D Systems) [32], equine interferon-γ (IFNγ; Equine IFNγ Duoset; R&D Systems) [34] and equine tumor necrosis factor-α (TNFα; Equine TNFα screening kit; Thermo Fisher Scientific) [34]. Equine IL-6 was measured exactly as previously described [35]. ELISA plates were read spectrophotometrically on a microplate reader with Gen5 software (Synergy HT Multi-Mode, Biotek, Winooski, VT). Canine TNFα, IFNγ, IL-6 and IL-10 were determined using a canine cytokine magnetic bead panel (Milliplex Map, EMD Millipore, Billerica, MA) per manufacturer instructions and were read on a Bio-Plex 200 using Bio-Plex Manager 4.1.1 software (Bio-Rad Laboratories, Hercules, CA).

In some assays the cyclooxygenase (COX) inhibitor indomethacin was used to chemically block PGE_2_ production. Indomethacin was added to MLR assays during plating at a concentration of 10 μM (Sigma-Aldrich) exactly as previously described [32].

### Statistical Analysis

Results are presented as median and interquartile range. Data were analyzed using non-parametric Mann-Whitney-Wilcoxon t-test (GraphPad InStat version 3.06 for Windows, La Jolla, CA). *P* < 0.05 was considered statistically significant.

## Results

### Equine and Canine MSC Cultured in HYPERFlasks® Did not Display Altered Proliferative Capacity, Immune-Suppressive Qualities or Mediator Profile

#### Equine

**Fig. 1:**
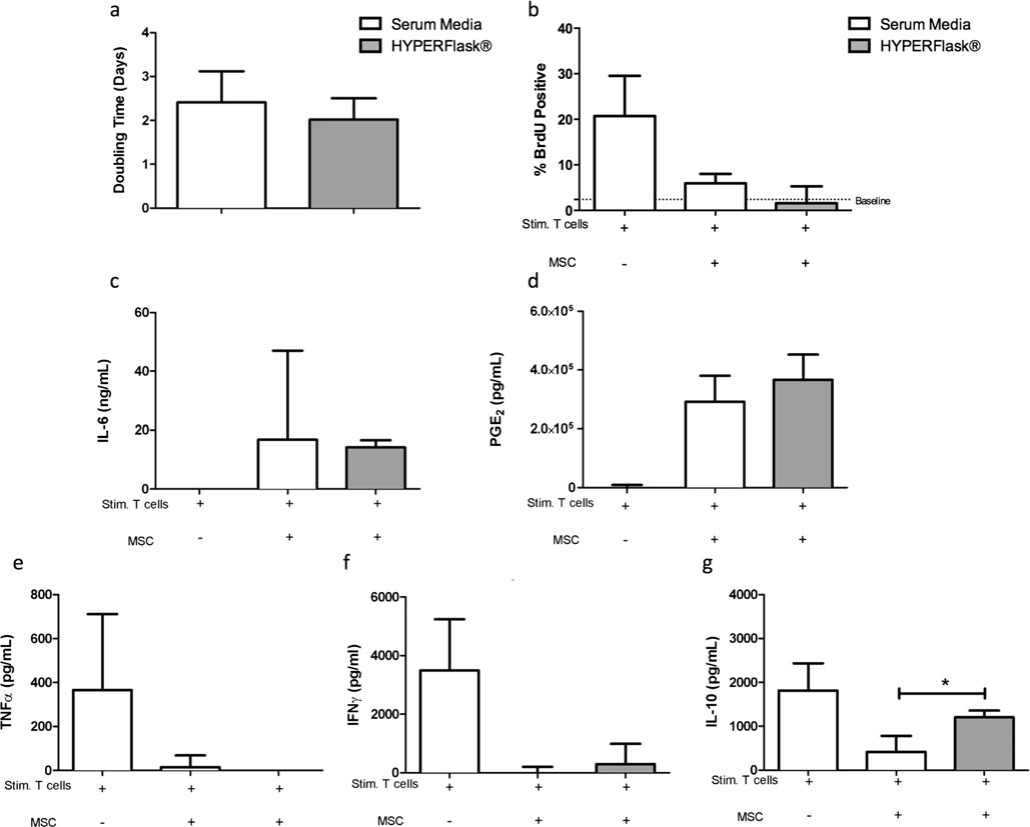
Equine MSC cultured in HYPERFlasks® did not display altered proliferative capacity, immune-suppressive qualities or mediator profile. No significant alterations in cell proliferation (population doubling time in days) of equine MSCs cultured in standard conditions were noted as compared to equine MSCs cultured in HYPERFlasks® (**a**). Similarly equine MSCs cultured in HYPERFlasks® maintain the ability to inhibit mitogen induced lymphocyte proliferation (**b**) and maintain a similar mediator profile as compared to MSCs cultured in standard conditions. HYPERFlask**®** cultured equine MSCs, in the presence of stimulated lymphocytes, produce IL-6 (**c**), PGE_2_ (**d**) and inhibit TNFα (**e**) and IFNγ (**f**) to the same degree as MSCs cultured in standard conditions. IL-10 production by stimulated lymphocytes however, was not reduced to the same amount as MSCs cultured in standard conditions (**g**). Data presented as a median and interquartile range

No alterations in the cell doubling times of eMSCs cultured in HYPERFlasks® (2.4 ± 0.3 days) were observed when compared to standard conditions (2.0 ± 0.6 days, Fig. 1a). However there was a marked increase in the absolute number of eMSCs produced after culture in HYPERFlasks®. This increase was due to the increased culture surface area (1721 cm^2^). Cells plated in HYPERFlasks® yielded ~4× more eMSCs than cells plated in standard conditions (44 × 10^6^ ± 24 × 10^6^ total cells compared to 9.3 × 10^6^ ± 5 × 10^6^ total cells).

We have previously published the immunomodulatory phenotype of equine MSCs cultured in standard conditions including results from MLR experiments [27, 32]. We found that equine MSCs inhibit activated lymphocyte proliferation via the induction of cell cycle arrest, inhibition IFNγ and TNFα secretion from activated T cells and the secretion of PGE_2_ and IL-6, among other mediators [27, 32]. The goal of the current project was to determine if this constellation of immune properties would be maintained after culture expansion in a more relevant cell expansion system and after expansion in SFM that did not contain FBS. Equine MSCs cultured in HYPERFlasks® maintained their ability to inhibit lymphocyte proliferation compared to eMSCs cultured in standard conditions (Fig. 1b). Mediator analysis of supernatants from MLR experiments demonstrated there was no difference in IL-6 (Fig. 1c) or PGE_2_ (Fig. 1d) secretion by activated eMSC cultured in HYPERFlasks® compared with eMSCs cultured in standard conditions. Equine MSCs grown in HYPERFlask® were as effective as eMSC cultured in standard conditions in inhibiting TNFα (Fig. 1e) and IFNγ (Fig. 1f) secretion by activated T-cells. However MSCs cultured in HYPERFlasks® secreted significantly higher concentrations of (*P* < 0.05) IL-10 (Fig. 1f) compared to eMSC cultured in standard conditions.

#### Canine

**Fig. 2:**
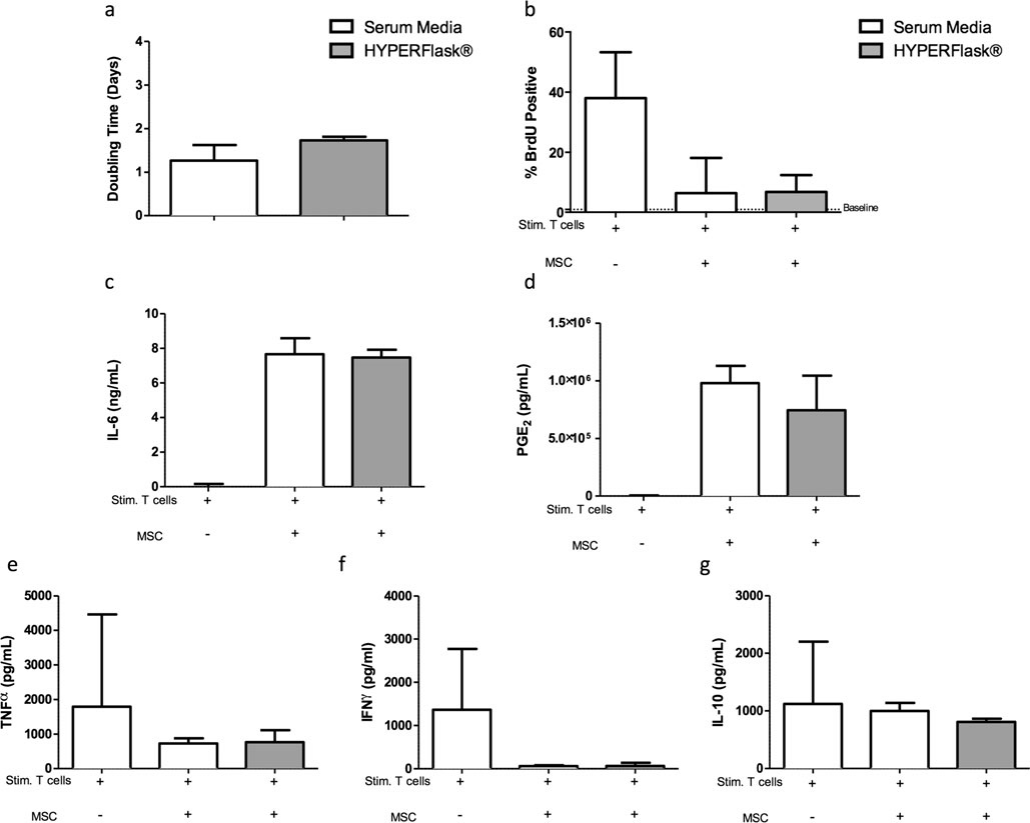
Canine MSC cultured in HYPERFlasks® also do not display altered proliferative capacity, immune-suppressive qualities or mediator profile. Canine MSCs cultured in HYPERFlasks® maintain similar proliferative rate (population doubling time in days) (**a**) and the ability to inhibit mitogen induced lymphocyte proliferation (**b**) as compared to canine MSCs in standard culture conditions. HYPERFlask**®** cultured canine MSCs, upon activation, produce IL-6 (**c**), PGE_2_ (**d**) and inhibit TNFα (**e**), IFNγ (**f**) and IL-10 (**g**) to the same degree as canine MSC cultured in standard conditions. Data presented as a median and interquartile range

As with eMSCs, we have previously published the immunophenotype of canine MSCs after culture in standard conditions in our laboratory [24, 36]. As such, we focused on if the culture expansion of cMSCs in a larger, more relevant flask system and in the absence of FBS would result in a different immunophenotype than standard culture. cMSCs cultured in HYPERFlasks® generally mirrored findings with eMSCs. Whereas cMSC doubling time was not altered when cultured in HYPERFlasks® (2 ± 0.6 days) compared with standard culture conditions (1.6 ± 0.2 days, Fig. 2a), HYPERFlasks® yielded ~18× more cells due to increased surface area (39 × 10^6^ ± 1.7 × 10^6^ total cells as compared to 2.3 × 10^6^ ± 6 × 10^6^ in two flasks cultured under standard conditions). cMSC cultured in HYPERFlasks® were as effective as cMSC cultured in standard conditions in inhibiting PBMC proliferation (Fig. 2b). There was no difference in the ability of cMSC to secrete IL-6 (Fig. 2c) and PGE_2_ (Fig. 2d) and to inhibit TNFα (Fig. 2e), IFNγ (Fig. 2f) and IL-10 (Fig. 2g) secretion by activated PBMCs for MSCs cultured in HYPERFlasks® compared to standard conditions.

### MSC Surface Phenotype is not Altered by Culture Conditions

#### Equine

In all culture conditions, eMSCs were positive for CD90, CD44 and CD29. Similarly, eMSCs did not express MHC II, CD86 or F6B (pan leukocyte marker) regardless of cell culture condition (Table 1).

**Table 1:** Surface protein markers as determined by flow cytometry

	Equine				Canine		
Marker	Standard (% +)	HYPERFIasks® (% +)	SFM (% +)	Marker	Standard (% +)	HYPERFIasks® (% +)	SFM (% +)
CD29	96 ± 0.1	95 ± 1.2	96 ± 1.2	CD54	97 ± 0.0	98 ± 0.0	97 ± 0.0
CD44	95 ± 0.7	95 ± 0.2	95 ± 1.1	CD44	98 ± 0.0	98 ± 0.0	99 ± 0.0
CD9O	83 ± 14.4	92 ± 1.9	88 ± 5.9	CD9O	91 ± 0.0	88 ± 0.0	96 ± 0.0
MHC II	0 ± 0.0	1 ± 0.6	4 ± 2.1	MHC II	0 ± 0.0	0 ± 0.0	0 ± 0.0
CD86	0 ± 0.0	0 ± 0.2	0 ± 0.0	CD34	1 ± 0.0	0 ± 0.0	1 ± 0.0
F6B	0 ± 0.0	0 ± 0.2	2 ± 0.9	CD45	0 ± 0.0	0 ± 0.0	0 ± 0.0

#### Canine

In all culture conditions, cMSCs were positive for CD44, CD54 and CD90. cMSCs did not express CD34, MHCII or CD45 (Table 1). Thus expansion of MSCs in either HYPERFlask® or in SFM did not alter standard cell surface protein expression in either eMSCs or cMSCs.

### MSC Proliferation Capacity is not Altered by Culture in SFM

#### Equine

**Fig. 3:**
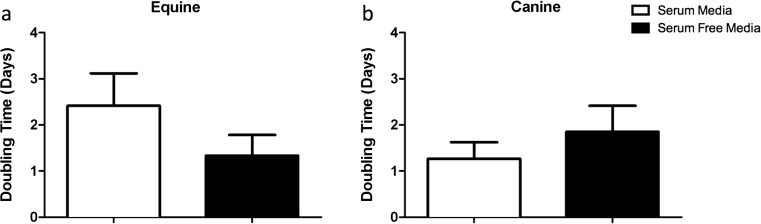
MSC proliferation capacity is not altered by culture in SFM. Cell proliferation (population doubling time in days) of eMSCs (**a**) and cMSCs (**b**) cultured in standard conditions and in SFM conditions. No significant alterations were found in cell doubling time between MSC cultured in standard and SFM conditions. Data presented as a median and interquartile range

eMSCs had population doubling times of 2.4 ± 0.3 and 2.4 ± 0.9 days in standard conditions and in SFM conditions, respectively (Fig. 3a). There was no statistical difference in doubling time of eMSCs between culture with and without FBS (*p* > 0.05).

#### Canine

The population doubling times for cMSCs were 1.4 ± 0.3 and 1.7 ± 0.8 days in standard conditions and in SFM conditions, respectively (Fig. 3b). There was no statistical difference in doubling time between cMSC cultured in standard media when compared to the SFM condition (*p* > 0.05).

### SFM Culture Conditions Alter eMSC Immunomodulatory Properties in Vitro

**Fig. 4:**
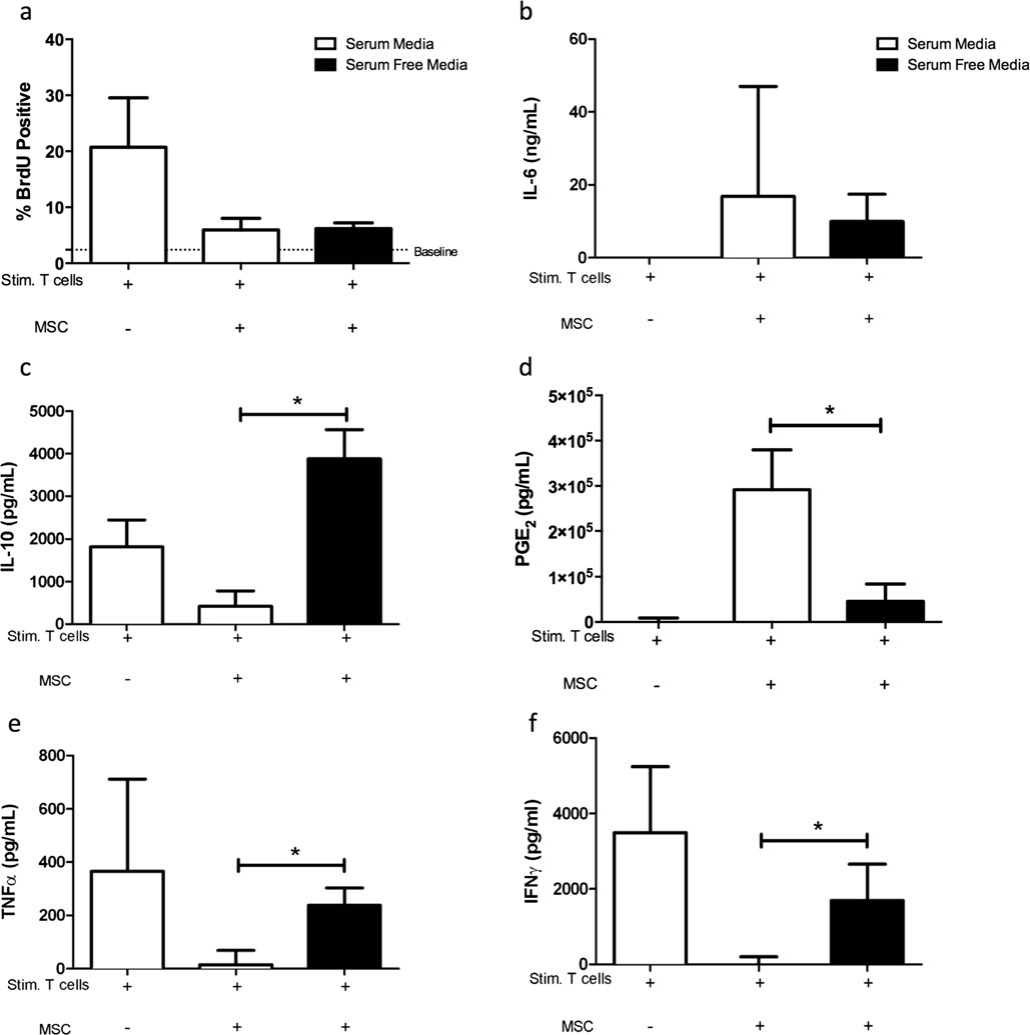
SFM culture conditions alter eMSC immunomodulatory properties in vitro. Stimulated lymphocytes were co-cultured with eMSCs in standard conditions and in SFM. A BrdU incorporation assay was used to record lymphocyte proliferation. Baseline unstimulated lymphocyte proliferation is indicated as a *dashed line*. No significant alterations were observed in the ability of eMSC to inhibit mitogen induced lymphocyte proliferation (**a**). MSC secretion of IL-6 was not altered by culture in serum free media (**b**). However, levels of IL-10 (**c**) and production of PGE_2_ (**d**) by activated MSCs in SFM was significantly altered in an inverse fashion, as compared to MSCs cultured in standard conditions. Inhibition of the pro-inflammatory mediators TNFα (**e**), and IFNγ (**f**) by stimulated lymphocytes was significantly altered in MSC cultured in SFM conditions. Data are presented as a median and interquartile range. *Bars with an asterisk* indicate significant differences in median values when compared to MSCs cultured in standard conditions (*p* < 0.05, Mann-Whitney-Wilcoxon)

While SFM-eMSC were as effective as eMSC cultured in standard conditions in their capacity to inhibit T-cell proliferation in vitro (Fig. 4a), secreted protein concentrations in MLR supernatant were altered when SFM-eMSC were compared with eMSC cultured under standard conditions. While IL-6 secretion by activated MSC (Fig. 4b) was not altered by eMSC culture method, the secretion of IL-10 (Fig. 4c) and PGE_2_ (Fig. 4d) displayed reciprocal inverse changes. IL-10 secretion was markedly and significantly (*P* < 0.05) increased when activated T-cells were co-cultured with eMSCs grown without FBS however PGE_2_ concentration was markedly and significantly (*P* < 0.05) decreased. Moreover, the concentrations of the pro-inflammatory mediators, TNFα (Fig. 4e) and IFNγ (Fig. 4f) were significantly higher when activated T-cells were co-cultured with eMSCs cultured in the absence of FBS compared to eMSCs cultured with FBS.

### SFM Culture Conditions Alter cMSC Immunomodulatory Properties in Vitro

**Fig. 5:**
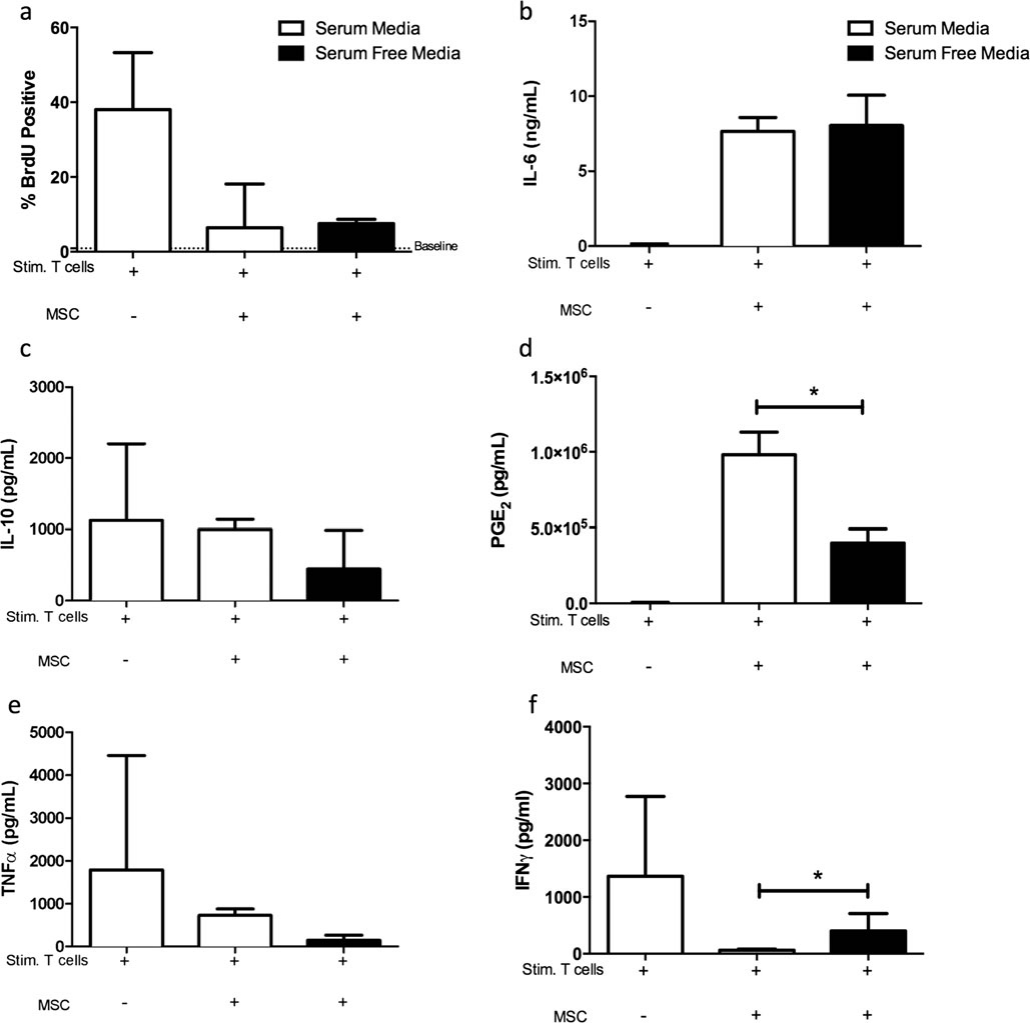
SFM culture conditions alter cMSC immunomodulatory properties in vitro. Stimulated lymphocytes were co-cultured with cMSCs in standard conditions and in SFM. A BrdU incorporation assay was used to record lymphocyte proliferation. Baseline unstimulated lymphocyte proliferation is indicated as a *dashed line*. No significant alterations were observed in the ability of cMSCs to inhibit mitogen induced lymphocyte proliferation (**a**). MSC secretion of IL-6 was not altered by culture in serum free media (**b**). Similarly, levels of IL-10 were also unaltered in MSC co-cultures in standard and SFM conditions (**c**). PGE_2_ production by activated cMSCs in SFM was significantly decreased as compared to MSC cultured in standard conditions (**d**). No changes were observed in inhibition of TNFα (**e**), however a significant increase was observed in levels of IFNγ (**f**) in MSC co-culture in SFM conditions. Data are presented as a median and interquartile range. *Bars with an asterisk* indicate significant differences in median values when compared to MSCs cultured in standard conditions (*p* < 0.05, Mann-Whitney-Wilcoxon)

Similar to eMSCs, cMSCs cultured in the absence of FBS were as effective in inhibiting PBMC proliferation in vitro as cMSCs grown under standard conditions. However, as in eMSCs, secreted protein concentration within the supernatants of these MLR experiments was altered by culture method. IL-6 and IL-10 secretion by activated cMSCs was not changed by the absence of FBS (Fig. 5a, b). However PGE_2_ secretion by activated cMSCs was markedly and significantly (*P* < 0.05) decreased in cMSCs cultured in the absence of FBS compared with cMSCs cultured in standard conditions. cMSCs cultured without FBS were similar in the ability to modulate TNFα secretion (Fig. 5e) by Con-A activated PBMC, compared to cMSCs in standard conditions. However IFNγ concentration was markedly and significantly (*P* < 0.05) higher when activated PBMCs were co-cultured with cMSCs in the absence of FBS.

### MSC-Derived PGE_2_ Modulated Equine Lymphocyte Proliferation but was not Responsible for the Inhibition of Canine Lymphocyte Proliferation

**Fig. 6:**
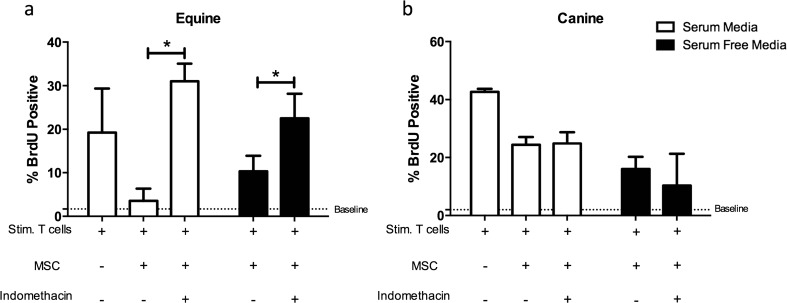
Inhibition of PGE_2_ synthesis restores equine lymphocyte proliferation but does not restore canine lymphocyte proliferation. Blocking of PGE_2_ production by equine (**a**) and canine (**b**) MSCs using the COX inhibitor indomethacin in a mixed leukocyte reaction setting. Blocking equine PGE_2_ restored T-cell proliferation in both standard and SFM conditions (*p* < 0.05, **a**). Blocking PGE_2_ production by canine MSCs did not restore PBMC proliferation in both standard and SFM conditions (**b**). Data are presented as a median and interquartile range. *Bars with an asterisk* indicated a significant change in the median of %BrdU positive lymphocytes as compared to standard MLR conditions (*p* < 0.05, Mann-Whitney-Wilcoxon)

We have previously reported that activated eMSCs from a variety of tissue sources inhibit T lymphocyte proliferation through a predominantly PGE_2_ mediated pathway [32]. This was determined through the use of indomethacin, a COX inhibitor, to block PGE_2_ synthesis and secretion. Given that eMSCs and cMSC cultured in the absence of FBS retained their capacity to inhibit lymphocyte proliferation in spite of secreting significantly less PGE_2_, we determined if PGE_2_ still mediated the inhibitory effect in MSCs cultured in SFM. In agreement with previous findings, blocking PGE_2_ production in eMSCs restored lymphocyte proliferation in standard conditions (Fig. 6a, *p* < 0.05). Similarly, blocking PGE_2_ synthesis in eMSCs cultured in the absence of FBS also inhibited T-cell proliferation (Fig. 6a, *p* > 0.05) suggesting that while the secretion of PGE_2_ is markedly decreased by eMSCs cultured without FBS, it is still the primary soluble factor that facilitates the inhibition of equine T-cell proliferation. In this study, blocking PGE_2_ synthesis by cMSCs did not restore lymphocyte proliferation in standard or SFM conditions (Fig. 6b).

## Discussion

In this study we determined the immunomodulatory and phenotypic consequences of a high yield, high performance tissue culture flask and FBS-free culture conditions for the culture of canine and equine MSCs. Both animal species are of great veterinary and translational research significance [7]. We determined that the expansion of canine and equine MSCs in HYPERFlasks® did not adversely alter MSC phenotype or immunomodulatory function and markedly improved MSC yield by providing 4–18× more MSCs in the same amount of time as traditional culture methods. This increase in MSC yield will allow more efficient banking of cells for research and clinical use. These findings are consistent with human MSC literature and may suggest that expansion systems are suitable for large scale and GMP-grade clinical use [21, 37, 38]. While optimal dosing of MSC for clinical applications has not been fully defined in literature, there is evidence that MSCs may act in a dose response fashion [20]. Large upscale production and banking of MSC will provide an accessible off the shelf product that may potentially be used for high dose clinical use pending further dose response studies.

Although the expansion of cMSCs and eMSCs in the absence of FBS did not alter MSC proliferation, surface phenotype or their capacity to inhibit T-cell proliferation in vitro, MSC immunomodulatory properties were significantly altered. It is important to note that while serum was removed from the SFM condition, bovine fibronectin was used as the minimal foreign component to allow for MSC adherence. Fibronectin is an extracellular matrix glycoprotein involved in cell adherence. Given MSC proliferation was not alerted in SFM conditions, fibronectin likely had no significant effects on MSCs in culture. Both cMSCs and eMSCs expanded in SFM secreted significantly less PGE_2_ than cells grown in FBS-containing media. cMSCs and eMSCs were also less able to inhibit pro-inflammatory cytokine production. These findings are concerning as PGE_2_ is a key mediator by which MSCs exert many of their immunomodulatory and trophic effects [1, 2, 39]. Prior to the removal or substitution of FBS from culture systems, a clearer understanding of how species-specific MSCs activate, signal and secrete their trophic and immunomodulatory mediators will be critical.

In our experiments, IL-10 concentration was inversely related to PGE_2_ concentration in eMSCs. Notably, eMSC cultured in HYPERFlasks® also significantly increased IL-10 production. These observations prompted us to hypothesize that in the relative absence of PGE_2_ secretion by activated MSCs, an alternative cellular pathway could be engaged which results in increased secretion (likely by activated T regulatory cells) of the potent immunoregulatory cytokine, IL-10. We further hypothesized that while the inhibition of PGE_2_ synthesis in standard culture conditions will rescue lymphocyte proliferation, such a rescue will not be seen in SFM conditions. Our results demonstrate that while IL-10 secretion was markedly induced in SFM conditions, PGE_2_ was still the primary effector mediator inhibiting equine lymphocyte proliferation, suggesting that a basal level of PGE_2_ in an MLR setting is sufficient to induce an inhibitory effect on T-cell proliferation. A similar response to PGE_2_ blocking was not seen in cMSCs, suggesting that PGE_2_ may play a less significant role in inhibiting lymphocyte proliferation in this species. These findings are consistent with previous studies in our laboratory [24] but are in disagreement with other studies that indicate the necessary role of PGE_2_ for cMSC mediated immune regulation [40, 41]. These discrepancies may be explained by differing methodologies, variable enrichment of T-cells versus PBMCs and perhaps MSC tissue source. Most notably, in our hands, cMSCs co-cultured with stimulated PBMCs produce nearly 10× the levels of PGE_2_ as compared to previous reports [40, 41]. Results from this in vitro study suggest that PGE_2_ secretion is not the primary mechanism by which MSCs mediate immunomodulation in dogs. An in-depth investigation of the role that MSC-derived PGE_2_ plays in lymphocyte regulation in dogs is warranted.

In this study, we discovered a disconnect between the inhibition of lymphocyte proliferation and a corresponding decrease in the proinflammatory mediators IFNγ and TNFα typically noted in a MLR [24, 32]. MSCs cultured in SFM markedly inhibited lymphocyte proliferation however the secretion of the pro-inflammatory mediators IFNγ (canine and equine) and TNFα (equine) was poorly inhibited. These data further suggest that in the absence of serum in the culture media, MSC immunomodulatory properties are compromised. The use of SFM for human MSC culture has remained controversial [11]. There have been reports that human MSCs can readily be expanded in SFM, while other groups argue that for MSCs to be expanded in serum free conditions, cytokines and other growth factors must be supplemented [11]. Our data coincides with previous reports, which suggest that while MSCs can proliferate in serum free conditions, sustained immunomodulatory properties in vitro are most potent in the presence of FBS [42–44].

The objectives of this study were to investigate potential modifications to ex vivo MSC expansion that would increase MSC yield and potentially permit removal of FBS to decrease the risk of FBS-induced immune response and culture heterogeneity. Our results suggest that HYPERFlask® provide a suitable scale up alternative to standard MSC culture, while substantially minimizing technician time and reagents required for culture of larger MSC doses. We also demonstrated that MSCs can be expanded successfully to achieve higher absolute cell numbers using HYPERFlask® without altering basic MSC characteristics including proliferation, phenotype or immunomodulatory functions. It can also be concluded that while canine and equine MSCs can be successfully cultured in a commercially available SFM media that does not alter surface phenotype or proliferative capacity, differing results in the mediator secretion profile warrant further investigation before the use of this media should be recommended for use in the clinical setting.

In conclusion, both eMSCs and cMSCs can be cultured in HYPERFlask® vessels to generate greater absolute numbers of MSC without altering MSC characteristics. Culturing MSCs in a commercially available SFM did not alter MSC proliferative capacity, surface protein phenotype or immune functions, however the cytokine profile was drastically altered. These findings warrant further studies for the use of xeno-free mediums for the culture of veterinary MSCs. These results also highlight species differences of MSCs and the need to further elucidate changes in mechanisms by which MSCs downregulate inflammation in different species.

*ACD* Acid-citrate dextrose, *BrdU* Bromodeoxyuridine, *cMSC* Adipose derived canine mesenchymal stem cell, *DMEM* Dulbecco’s Modified Eagle Medium, *DPBS* Dulbecco phosphate buffered saline, *ELISA* Enzyme-linked immunosorbent assay, *eMSC* Bone marrow derived equine mesenchymal stem cell, *FBS* Fetal bovine serum, *GMP* Good manufacturing practice, *IFNγ* Interferon-γ, *MSC* Mesenchymal stem cell, *PBMC* Peripheral blood mononuclear cells, *PGE*_*2*_ Prostaglandin E_2_, *SFM* Serum free media, *TNFα* Tissue necrosis factor α, *UCD* University of California, Davis
